# Awareness of Sensorimotor Adaptation to Visual Rotations of Different Size

**DOI:** 10.1371/journal.pone.0123321

**Published:** 2015-04-20

**Authors:** Susen Werner, Bernice C. van Aken, Thomas Hulst, Maarten A. Frens, Jos N. van der Geest, Heiko K. Strüder, Opher Donchin

**Affiliations:** 1 Institute of Movement and Neurosciences, German Sport University, Cologne, Germany; 2 Department of Neuroscience, Erasmus MC, Rotterdam, The Netherlands; 3 Erasmus University College, Rotterdam, The Netherlands; 4 Department of Biomedical Engineering, Ben-Gurion University of the Negev, Beer-Sheva, Israel; Duke University, UNITED STATES

## Abstract

Previous studies on sensorimotor adaptation revealed no awareness of the nature of the perturbation after adaptation to an abrupt 30° rotation of visual feedback or after adaptation to gradually introduced perturbations. Whether the degree of awareness depends on the magnitude of the perturbation, though, has as yet not been tested. Instead of using questionnaires, as was often done in previous work, the present study used a process dissociation procedure to measure awareness and unawareness. A naïve, implicit group and a group of subjects using explicit strategies adapted to 20°, 40° and 60° cursor rotations in different adaptation blocks that were each followed by determination of awareness and unawareness indices. The awareness index differed between groups and increased from 20° to 60° adaptation. In contrast, there was no group difference for the unawareness index, but it also depended on the size of the rotation. Early adaptation varied between groups and correlated with awareness: The more awareness a participant had developed the more the person adapted in the beginning of the adaptation block. In addition, there was a significant group difference for savings but it did not correlate with awareness. Our findings suggest that awareness depends on perturbation size and that aware and strategic processes are differentially involved during adaptation and savings. Moreover, the use of the process dissociation procedure opens the opportunity to determine awareness and unawareness indices in future sensorimotor adaptation research.

## Introduction

The involvement of cognitive components such as explicit strategies and explicit knowledge, i.e. awareness, in motor learning has been intensively investigated since their manipulation is thought to lead to beneficial effects on various types of motor learning [1–3], and could thus be used in rehabilitation programs or athletes training schedules. In sensorimotor adaptation, learning was found to be increased in participants with explicit knowledge compared to those without [4]. In that study, participants performed reaching movements to visual targets while either adapting to a clockwise (CW) or a counter-clockwise (CCW) force field acting on the moving arm. Those participants, who were aware of the specific pattern of perturbation, showed a larger learning index. However, the calculation of this learning index did not allow a distinction between improvements during adaptation phase and adaptive recalibration of the sensorimotor system, which is commonly measured by reaches made without visual feedback during (catch trials) or after training (aftereffects) [1, 5]. More specifically, explicit knowledge has a positive effect on the adaptation phase but not on recalibration, as indicated by the transfer to a new motor task in a further study [6]. In addition, numerous studies showed an age-related impairment of sensorimotor adaptation during adaptation phase but not during aftereffect tests [7–10, 5] and this impairment of adaptation correlated with age-related reduction of explicit knowledge [7, 11, 12]. To sum up, these results indicate a beneficial effect of explicit knowledge on performance during adaptation phase, but not on recalibration.

In the aforementioned studies, sensorimotor perturbations were induced suddenly leading to large movement errors at the beginning of adaptation. When perturbations are introduced gradually in a stepwise fashion, only small movement errors occur. Compared to sudden adaptation, performance in aftereffect tests was improved after gradual prism [13] and saccade adaptation [14], after gradual adaptation to a visual gain [15] and to a 90° rotation [16]. Adaptation to smaller visual rotations (30° and 60°), however, did not lead to a difference of aftereffects between sudden or gradual adaptation [17, 18]. The difference of recalibration between both conditions is usually associated with a difference in awareness of the knowledge acquired through adaptation [1, 19, 15, 20, 21].

Thus, there is a contradiction between studies showing enhanced explicit knowledge not effecting recalibration as shown in the first section and others claiming that unawareness, i.e. absent explicit knowledge, results in an increase of recalibration as shown in the second section. One explanation of this inconsistency could be an intermingling effect of explicit strategies, which might have been applied independently by some participants. Explanations of the nature of the perturbation or strategies like deliberate past pointing were shown to lead to a faster reduction of errors [21–23, 1], but also to lessened aftereffects [23, 1] compared to adaptation without explicit knowledge. Another explanation could be the dependence of explicit knowledge on the size of the perturbation, as previous results suggest: Hegele and Heuer showed that elderly participants made less use of explicit instructions than younger participants during adaptation to a large visual rotation of 75° [24], whereas cerebellar patients were able to successfully apply an explicit strategy of reaching to aiming targets, which counteracted a smaller visual rotation of 45° [25]. While cerebellar patients clearly suffer from cerebellar atrophy, anatomical evidence also reveals an age-related atrophy of the cerebellum [26–29]. The different results could, thus, be related to a difference of affected anatomical regions, but also to a difference of rotation sizes. Furthermore, different generalization patterns to untrained targets were found after adaptation to a 75° [30, 12] and to a 30° rotation [31]. To our knowledge, awareness has as yet not been measured after different perturbation sizes.

Two further drawbacks of previous research on awareness or explicit knowledge during motor adaptation can be identified. The first drawback is the irregular use of the term awareness. Some authors refer to awareness of a perturbation, i.e. the notion that something has changed [20, 21]. Others more specifically mean the awareness of the nature of the perturbation [32, 1], as we relate to in the present study. The second drawback is the widespread use of questionnaires as a means of measuring awareness [4, 1, 6, 33]. In some studies using the gradual adaptation paradigm, even a formal questionnaire was not used. As discussed below, these methodologies have several disadvantages.

The domain of cognitive psychology has a strong tradition of analysing awareness and there is an enduring debate about the acquisition of knowledge and whether or not it is available to conscious access [34–38]. Questionnaires were soon the target of criticism in this field since verbal responses may fail to exhaustively reveal all of a subjects’ unconscious knowledge because the knowledge is weak or held with low confidence, on the one hand, or because of the sharp difference of retrieval contexts (motor response vs. verbal response), on the other hand [39, 38, 40, 41, 37]. Alternative methods largely abandoned in cognitive research are prediction tasks during which participants are asked to make predictions during the same sort of task, to generate the same task freely or to recognize the trained task after an adaptation phase [42–44]. Those methods have recently been implemented in sensorimotor adaptation research [11, 12, 45, 21] but they suffer from the disadvantage that performance might be based on feelings of familiarity [46–49] and might, therefore, lead to an overestimation of awareness [36, 37]. Consequently, cognitive psychologists developed a method called the process dissociation procedure (PDP). First used by Jacoby (1991), PDP is widely accepted and used today in the cognitive domain [50–55, 34, 56]. Based on the assumption that conscious knowledge is controllable, aware and unaware learning can be estimated by comparing performance when subjects attempt to either express or repress the learned behavior.

The present project pursued the idea of incorporating the current best methodology for measuring awareness and unawareness into the study of sensorimotor adaptation. We decided to test whether the degree of awareness depends on the magnitude of the perturbation applied to the subjects´ movements and whether it can be manipulated by providing the participants with an explicit strategy before adaptation.

## Materials and Methods

### Participants

Twenty-four right-handed subjects participated in the study in exchange for course credit and were randomly assigned to an implicit (n = 16; age: 20.2 ± 3.4; 14 female) or an explicit group (n = 8; age 21.0 ± 3.2; 8 female). Participants’ ages ranged from 18 to 32 years, thus all reaching majority in the country of testing. The participants of the first group were told that there would be a perturbation, but remained uninformed about its nature. The participants of the latter group, however, received detailed explanations with the help of a clock face as in Benson et al. (2011). None of the subjects had any experience in visuomotor adaptation research or exhibited overt sensorimotor deficits besides corrected vision. The experimental protocol was pre-approved by the Erasmus MC Medical Ethical committee, was conducted according to the principles expressed in the Declaration of Helsinki and all subjects gave written informed consent.

### Task

**Fig 1:**
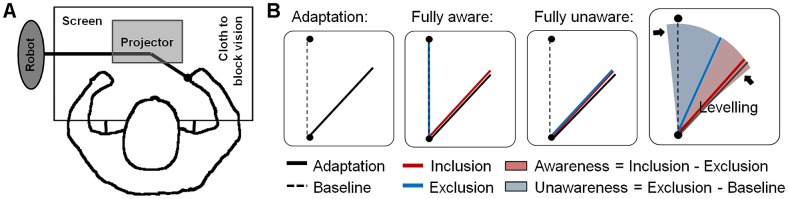
Scheme of experimental apparatus (A). Shown are robot, display screen and projector. Measuring awareness and unawareness (B). Exemplary adaptation, inclusion and exclusion movement directions indicating fully aware or unaware behaviour. Schematic and simplified presentation of awareness and unawareness. Note that for calculation of an awareness and unawareness index normalized mean movement directions of inclusion and exclusion were used in order to allow comparison between rotation angles (see Methods). Movement directions were levelled between baseline direction -10% and size of perturbation +10% as indicated by the arrows.

Participants watched a horizontal screen and held the handle of a robot that was placed underneath the screen, while a cloth prevented the sight of their arm (Fig 1A). The position of the robot-handle was registered with a sampling frequency of 200 Hz and a resolution of 0.3×10^-3^ degrees on each joint of the shoulder which translates into a resolution in Cartesian coordinates of less than 0.2 mm. The registered signal was used to display a green cursor (diameter 6 mm) representing the handle position onto the screen with the help of a projector. Furthermore, a black origin and one of three possible red targets were alternately projected. The origin was positioned approximately 45 cm away from the eyes of the participant and the targets were positioned 10 cm away from the origin, either straight ahead or 45° to the left or the right. The origin as well as the targets had a diameter of 14 mm and the cursor one of 6 mm. The subjects were instructed to move the cursor accurately and quickly from the origin to the target and back. To control for the speed of the movements target color changed to green for actual trail times of 850ms ± 100ms and turned to blue or yellow when movements were too slow (>950ms) or too fast (>750ms), respectively. Intertrial intervals lasted for 1500ms.

After *familiarisation* with veridical and *baseline without visual feedback*, i.e. no cursor visible, all participants conducted six sets, each containing a *baseline*/*washout* block with veridical visual feedback, an *adaptation* block with rotated visual feedback (20°CW, 40°CW or 60°CW) and an *inclusion* and *exclusion* block without feedback. During each adaptation block, six clamp trials were inserted to test for the progression of recalibration (trial number 6, 19, 30, 39, 47 and 58). In those trials a perfect movement of the cursor from the starting to the target dot was displayed independent of the subjects´ movement. Each participant performed two consecutive sets for each rotation size with alternating order of inclusion and exclusion blocks. Before inclusion subjects were instructed to ‘use what was learned during adaptation’ and before exclusion subjects were asked to ‘refrain from using what was learned, perform movements as during baseline’. This order as well as rotation size order was randomised between participants. Between the third and fourth set there was a rest break of 5 min. Table 1 shows an overview of the experimental protocol.

**Table 1:** Experimental protocol.

	Blockname	# of Trials	Visual FB
**Intro**	Familiarization	6	0°
	Baseline no FB	6	-
**Set 20**	Baseline/Washout	60	0°
	Adaptation	60	-20°
	Exclusion/Inclusion	9	-
	Inclusion/Exclusion	9	-
**Set 40**	Baseline/Washout	60	0°
	Adaptation	60	-40°
	Exclusion/Inclusion	9	-
	Inclusion/Exclusion	9	-
**Set 60**	Baseline/Washout	60	0°
	Adaptation	60	-60°
	Exclusion/Inclusion	9	-
	Inclusion/Exclusion	9	-

Visual feedback (FB) was either not present (-), veridical (0°) or rotated (20°, 40° or 60°). Each set was performed twice with alternating order of exclusion and inclusion.

After completion of the experiment all participants filled out a questionnaire as in Benson et al. (2011). Those participants who characterized the perturbation as a rotation or reported the use of a rotational strategy were considered to be explicitly aware of the distortion.

### Data processing

After the conclusion of the experiment, movement direction (MD) was determined as the angle between a line connecting starting and target dot and a line between movement onset and movement position at 150 ms after movement onset. Movement onset was defined as the movement position at which velocity exceeded 0.03 mm/ms for the first time. The trial was omitted if the distance between movement onset and movement position at 150 ms after movement onset was smaller than 10 mm; 4.59% of trials were thus excluded.

From the movement directions of each subject and in order to compare the three rotation sizes, we calculated normalized indices for the different parameters. Adaptation index (AI) and the clamp trial index (CI) were determined as

$$AI\;=\;\frac{{MD}_{adaptation\;trials}-{MD}_{baseline\;trials}}{Rotation\;size-{MD}_{baseline\;trials}}$$ (1)

and

$$CI\;=\;\frac{{MD}_{clamp\;trials}-{MD}_{baseline\;trials}}{Rotation\;size-{MD}_{baseline\;trials}}$$ (2)

Where *MD*_*clamp trials*_ and *MD*_*adaption trials*_ were calculated as the mean of the MD for the trials in a specific bin of trials. We used bins of 9 trials for adaptation trials and bins of 3 trials for clamp trials. We calculated *MD*_*baseline trials*_ as the mean MD of all baseline trails. Both indices range from -1 to 1 with 1 indicating full adaptation and 0 (or a negative number) indicating no adaptation. If a subject produced an AI of ≤ 0.2 during the last two bins of an adaptation block, data for that set was dropped from further analysis. Thus, for each rotation size the data of three participants was excluded (20°: two implicit subjects and one explicit; 40° and 60°: three implicit subjects each). This resulted in a total amount of 12 implicit and 7 explicit subjects.

During the inclusion condition awareness and unawareness both contribute to performance. In the exclusion condition, however, aware and unaware learning are set in opposition. If all knowledge acquired through adaptation is conscious, i.e. a person is fully aware, performance during the exclusion task (E) should not be different from performance during baseline (B). Or, inversely, E > B can be seen as evidence for unaware knowledge. If, in addition, performance during the inclusion task equals that during exclusion, the person is fully unaware (Fig 1B). Within the PDP, an estimate of awareness can therefore be derived from the difference between exclusion and inclusion performance and an estimate of unawareness can be obtained from the difference between exclusion performance and baseline as shown schematically in Fig 1B [35]. Thus, we first calculated exclusion and inclusion indices (EI and II) as

$$EI\;=\;\frac{{MD}_{exclusion\;trials}-{MD}_{baseline\;no\;FB\;trials}}{{MD}_{last\;adaptation\;trials}-{MD}_{baseline\;trials}}$$ (3)

and

$$II\;=\;\frac{{MD}_{inclusion\;trials}-{MD}_{baseline\;no\;FB\;trials}}{{MD}_{last\;adaptation\;trials}-{MD}_{baseline\;trials}}$$ (4)

Again we chose a bin size of nine consecutive trials for exclusion, inclusion and the last adaptation trials and mean values of all trials were used for both baseline blocks. In the original publication introducing the PDP the inclusion and exclusion tests asked for a classification of words into ‘new’ or ‘old’ depending on its appearance in previous phases of reading, hearing or solving of anagrams of words [47]. Thus, answers were dichotomous. In contrast, the present inclusion and exclusion conditions allow for an answer, i.e. movement direction, continuously ranging from reproducing the learned movement to reproducing baseline movements. And, because of the circular nature of possible movement directions, even movement directions beyond the learned movement direction or beyond baseline direction could occur. In order to retain as much information as possible without allowing outliner movements to weight results, we decided to level all movement directions of exclusion and inclusion blocks between the rotation size of the previous adaptation block plus 10% and mean baseline movement direction minus 10% (Fig 1B). For example, movement directions in an exclusion or inclusion block that followed adaptation to 20°CW rotation should ideally range from -20° to 0°. During levelling all smaller or larger movement directions (cut off value 180°) were levelled to -22° or 2°, respectively. Finally, awareness and unawareness were calculated from EI and II with the awareness index equalling II minus EI and the unawareness index equalling EI.

For statistical analysis we submitted the different indices to several analyses of variance (ANOVAs) with the between-factor Group (implicit, explicit) and the within-factors Rotation Size (20°, 40°, 60°), Block Order of inclusion and exclusion (first, second) and Bin. Type III sums of squares were used in all analyses unless stated otherwise. Normality within each Bin, Rotation Size and Block Order in each group separately was explored by Shapiro-Wilk test and variance stability of the pairs of levels across the factor Group was explored by Levene´s test. In case of violation, a Kruskal-Wallis-Test with the factor Group (explicit, implicit) was performed with the respective data point. Greenhouse-Geisser-adjustments were applied when necessary to compensate for heterogeneity of variances and significant effects were further explored with Turkeys HSD post-hoc tests for unequal sample sizes. Furthermore, Pearson product-moment correlation coefficients (PCC) between awareness and adaptation indices were calculated in case of normality and variance stability of the respective data. Otherwise Spearman's rank correlation coefficients (SCC) were used. All analyses were done with Statistica 7.1.

## Results

**Fig 2:**
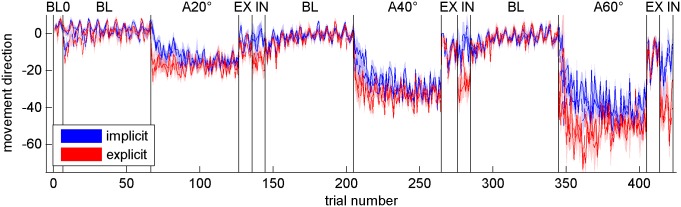
Movement directions of all trials. Shown are movement directions with respect to target direction of implicit (blue) and explicit (red) participants for all experimental phases. Symbols indicate across-subject means, and the shaded area display standard errors. Note that rotation size order was randomised between participants.

Fig 2 depicts the mean movement directions of all trials of the implicit and explicit group. Note that the shown order of 20°, 40°, and 60° is only exemplary, since rotation size order was randomised between participants. Due to randomizations of inclusion and exclusion block order each rotation size was consecutively performed twice. Clearly from this presentation, the performance of both groups was very similar during all baseline phases. Absolute movement direction angles gradually increased during each adaptation block with larger movement direction angles at the end of adaptation to 40° than to 20° rotation and during adaptation to 60° rotation movement direction angles were larger still. This was consistent across groups. During the beginning of each adaptation phase, however, the figure shows slightly larger movement direction angles for the explicit group. Movement directions of exclusion blocks abruptly return close to baseline level in both groups, whereas during inclusion blocks movement directions approach adaptation level more so in the explicit group.

**Fig 3:**
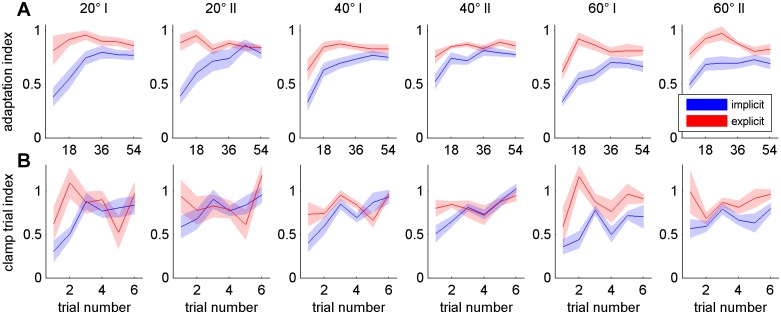
Adaptation and clamp trial indices. Mean adaptation (A) and clamp trial indices (B) of implicit (blue) and explicit (red) participants. The shaded area indicates standard errors. Note that a bin size of nine trialswas used for calculation of the adaptation index. For the clamp trial index bin size of one trial was used in this figure for illustrative purposes, but bin size of three trials was used for statistical analysis. For the adaptation index statistical analysis revealed significant effects of Group (p<0.01), Bin (p<0.001), Block Order (p<0.05), Bin × Group (p<0.001) and Bin × Block Order (p<0.01). For the clamp trial index the analysis yielded significant effects of Group (p<0.05), Bin (p<0.01), Block Order (p<0.05) and Bin × Group (p<0.01). Both analyses revealed no significant effects of Rotation Size or any other interaction (all p>0.05).

The above observations of behaviour during adaptation blocks are confirmed by statistical analysis of the adaptation index AI, which is shown in Fig 3A. An ANOVA with the factors Group (explicit, implicit), Rotation Size (20, 40, 60), Bin (1:6) and Block Order (I, II) yielded several significant effects. The adaptation index of the explicit group was larger than that of the implicit group (effect of Group (F(1,16) = 11.58; p<0.01)) and it increased during the course of adaptation (effect of Bin (F_corrected_(3,43) = 32.52; p<0.001)). We further found effects of Block Order (F(1,16) = 4.73; p<0.05), Bin × Group (F_corrected_(3,43) = 12.81; p<0.001) and Bin × Block Order (F_corrected_(4,60) = 3.80; p<0.01). Post-hoc decomposition of Bin × Group showed no significant difference of AI between both groups for each respective bin. However, AI of the first bin of the implicit group is different from all following bins of both groups and the second bin is different from the last three bins, whereas in the explicit group the first bin is only different from the second and third bin of this group. Hence, there was a larger AI during the beginning of adaptation in the explicit group (Fig 3A). In addition, post-hoc analyses of Bin × Block Order revealed that AI of the first bin is significantly different between the first and second run. Due to the counterbalanced order of inclusion and exclusion blocks this savings effect should have no effect on the analysis of those blocks. Seven data points out of 72 did not show a normal distribution (6x p<0.05, 1x p<0.01). Levene´s test revealed no homogeneity of variance for six out of 36 data points (5x p<0.05, 1x p<0.001). For all those data points we performed Kruskal-Wallis-Tests with the factor Group (explicit, implicit) and 7 out of 13 revealed a significant difference between the implicit and explicit group according to the results of the analysis of variances (3x p<0.05, 4x p<0.01).

Fig 3B shows the mean group values of all clamp trial indices. An analysis of variances of the clamp trial index with bin size 3 yielded a significant effect of Group (F(1,17) = 6.42; p<0.05) with a higher clamp trial index in the explicit than the implicit group. It also revealed significant effects of Block Order (F(1,17) = 5.75; p<0.05), Bin (F(1,17) = 15.38; p<0.01) and Bin × Group (F(1,17) = 8.82; p<0.01) with a significant difference between first and second bin in the implicit but not in the explicit group. Levene´s test revealed no homogeneity of variance for two out of twelve data points (both p<0.05). Kruskal-Wallis-Tests with the factor Group (explicit, implicit) confirmed a significant difference between groups for one of the two data points (p<0.01).

**Fig 4:**
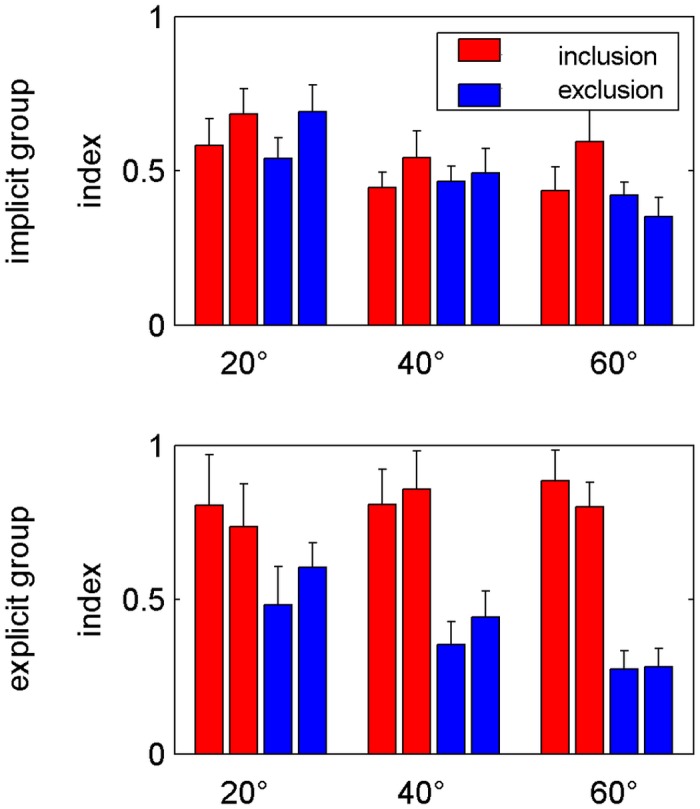
Inclusion and exclusion. Mean inclusion (red) and exclusion (blue) indices for all three perturbation sizes (20°, 40° and 60°) separately for the implicit (A) and explicit (B) group. Error bars indicate standard errors. For the inclusion index statistical analysis yielded a significant effect of Group (p<0.05), but no effects of Rotation Size, Block Order or any interaction (all p>0.05). For the exclusion index the analysis revealed a significant effect of Rotation Size (p<0.001). No significant effects of Group, Block Order or any interaction was found here (all p>0.05).

Fig 4 illustrates the inclusion and exclusion indices of both groups. The first and second block of each rotation size and group are shown for both conditions. Note that the first block directly succeeded an adaptation block but could be second in the order of the experimental protocol due to randomization of inclusion and exclusion blocks. While inclusion and exclusion indices show similar extents in the implicit group, a clear dissociation can be observed in the explicit group. Fig 4 further shows a decrease of the exclusion index from 20° to 40° to 60° adaptation. In addition, the inclusion index tended to be larger during the second block than during the first one, however, this was not consistent over rotation sizes or groups. Statistical analysis for the inclusion index revealed a significant effect of Group (F(1,17) = 6.35; p<0.05), whereas the analysis of the exclusion index only yielded an effect of Rotation Size (F(2,34) = 19.30; p<0.001). Here the exclusion index after adaptation to 20° rotation differed significantly from that after 40° and 60°, but there was no difference of the exclusion indices after 40° and 60° adaptation. No block order effects were revealed for both conditions supporting our earlier visual observation. The mean values of both blocks are thus used in the following analyses.

**Fig 5:**
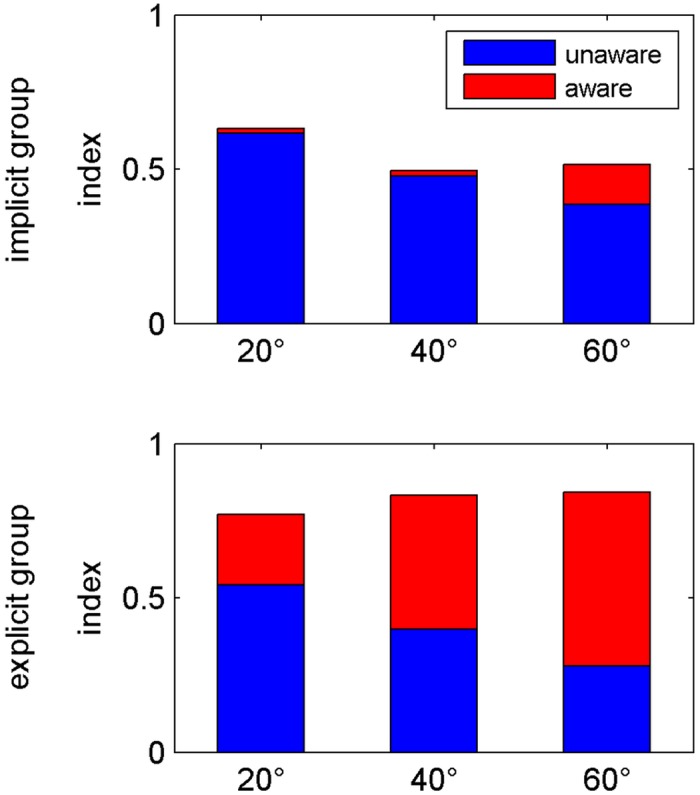
Awareness and unawareness. Mean awareness (red) and unawareness (blue) indices for all three perturbation sizes (20°, 40° and 60°) separately for the implicit (A) and explicit (B) group. For the awareness index statistical analysis revealed significant effects of Group (p<0.01) and Rotation Size (p<0.05), but no significant interaction (p>0.05). For the unawareness index the analysis yielded a significant effect of Rotation Size (p<0.001), but no significant effects of Group or Group × Rotation Size (both p>0.05).

The main results of the present study are shown in Fig 5. Awareness index is very low within the implicit group with only some awareness after adaptation to a 60° rotation. In the explicit group, however, Fig 5 reveals an increase of the awareness index with increasing rotation size and with an equal amount of awareness and unawareness index after adaptation to a 40° rotation. We performed two ANOVAs for aware and unaware each with the factors Group (explicit, implicit) and Rotation Size (20, 40, 60). The analysis of the awareness index revealed significant effects of Group (F(1,17) = 8.66; p<0.01) and Rotation Size (F_corrected_(1,24) = 6.20; p<0.05) with a difference of awareness of adaptation to 20° and 60°. The mean awareness index after 20° and 60° adaptation increased from 0.02 to 0.13 in the implicit and from 0.23 to 0.56 in the explicit group. Even though this increase was larger in the explicit group, we found no significant interaction of Group × Rotation Size. The unawareness index did not differ between both groups, but also did depend on the size of the rotation (Rotation Size: F(2,34) = 19.30; p<0.001) with post-hoc tests revealing a significant difference between 20° and both 40° and 60° but not between 40° and 60°.

It is possible that awareness could benefit from previous training with another rotation. Therefore, the analysis of the awareness index was repeated with the additional factor Rotation Size Order (20-40-60, 20-60-40, 40-60-20, 40-20-60, 60-20-40, 60-40-20). Type VI or *Effective Hypothesis* sums of squares were used in this ANOVA since not all individual groups were included due to missing data. We found no significant effects either of Rotation Size Order or of any interaction including this factor (all p>0.05). However, groups were not all normally distributed and group sizes were very small. To increase group sizes, we, thus, omitted the factor Group and calculated another analysis using only the factors Rotation Size and Rotation Size Order. This analysis also did not reveal a significant effect of Rotation Size Order or its interaction with Rotation Size (both p>0.05). Since two data points out of 18 did not show a normal distribution we calculated a Kruskal-Wallis-Test for each rotation size using the factor Rotation Size Order. None of them revealed a significant effect (all p>0.05).

**Fig 6:**
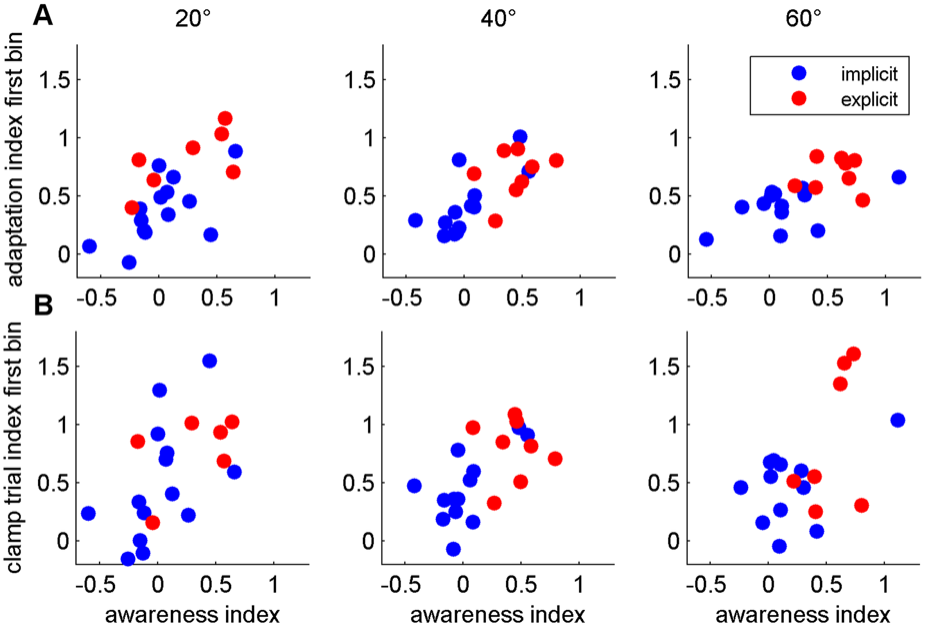
Correlations between awareness and adaptation. Correlations between the awareness indices for each rotation size and the respective adaptation (A) or clamp trial index (B). Red dots indicate explicit and blue ones implicit participants.

To compare the extent of awareness to the amount of adaptation for the different rotation sizes, we first calculated correlations between the awareness index and the mean adaptation and clamp trial index of the first bin of both blocks, respectively (Fig 6). Larger awareness of the learned perturbation was related to larger adaptation and clamp trial indices of the first bin. Second, we calculated correlations between the awareness index and the mean adaptation index of the last bin of both blocks. Here, we found a significant correlation for 40° rotation only. All results are presented in Table 2.

**Table 2:** Correlation of awareness and adaptation.

		Awareness index		
		20°	40°	60°
Adaptation index	first bin	0.76***	0.73***	0.51*
	last bin	0.32	0.60**	0.30
Clamp trial index	first bin	0.51*	0.70***	0.59**

Pearson product-moment correlation coefficients of the correlations between awareness and adaptation (first and last bin) or clamp trial indices (first bin) are shown, respectively. Symbols ***, **, and * indicate p<0.001, p<0.01, and p<0.05, respectively, and the absence of a symbol indicates p>0.05.

**Fig 7:**
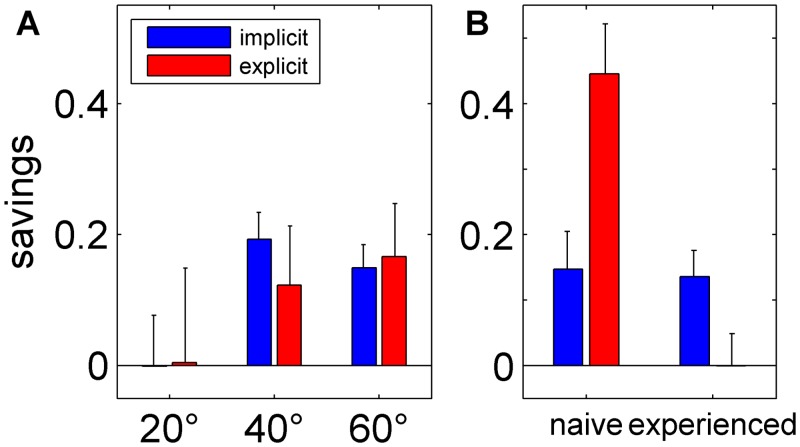
Savings. Mean savings for the implicit (blue) and explicit (red) group for all three perturbation sizes (20°, 40° and 60°) (A) as well as for naïve adaptation, i.e. the first rotation size of the experimental protocol, and experienced adaptation, i.e. the second and third rotation sizes (B). For the three rotation sizes statistical analysis revealed no significant effects (all p>0.05). An analysis of the naïve and experienced condition yielded significant effects of Group (p<0.05), Condition (p<0.05) and Group × Condition (p<0.01).

Measuring savings, i.e. faster relearning during a second exposure to the same perturbation, is a tool for determining whether a long-term memory of the adaptation has been established. We calculated savings as the initial learning difference score that is the difference of the adaptation index of the first bin of the first and the second block of adaptation to the same rotation size [57]. For both groups there were almost no savings in the 20° rotation and up to about 20% savings in the 40° and 60° rotation condition as shown in Fig 7A. ANOVA with the factors Group (explicit, implicit) and Rotation Size (20, 40, 60) further revealed no significant effects but a trend in Rotation Size (p = 0.06). Two data points out of six did not show a normal distribution (1x p<0.05, 1x p<0.01) and we, therefore, performed Kruskal-Wallis-Tests with the factor Group (explicit, implicit). According to the results of the analysis of variances no group effect was revealed (both p>0.05). We further found no correlation between the savings and awareness indices (20°: PCC = -0.22, p>0.05; 40°: PCC = 0.02, p>0.05; 60°: PCC = 0.39, p>0.05).

We were further interested whether savings also transferred across rotation sizes. Hence, naïve savings was determined using only the first rotation size of the experimental protocol and experienced savings was additionally calculated using the second and third rotation sizes. Note that this differed between participants; the first rotation size was 20°, 40° or 60° for eight participants each. While naïve savings was larger in the explicit than in the implicit group the reverse holds for experienced savings as can be observed in Fig 7B. The statistical analysis with the factors Group (explicit, implicit) and Condition (naïve, experienced) yielded significant effects of Group (F(1,17) = 4.79; p<0.05), Condition (F(1,17) = 8.23; p<0.05) and Group × Condition (F(1,17) = 9.14; p<0.01). Here, naïve savings of the explicit group was different from all other savings values as confirmed by post hoc analysis of the interaction. A normal distribution was not given for naïve savings of the implicit group (p<0.05). Kruskal-Wallis-Tests with the factor Group (explicit, implicit) confirmed the difference of naïve savings between groups (p<0.05). No Spearman correlations were found between naïve or experienced savings on the one hand and awareness indices of respective rotation sizes on the other (naïve: SCC = 0.24, p>0.05; experienced: SCC = -0.33, p>0.05).

Finally, we compared the present measurement of awareness by the PDP to that of a questionnaire. Therefore, the results of the verbal assessment were used to classify all subjects into aware or unaware according to the approach used by Benson et al. (2011). Within this procedure explicit awareness was attributed to those participants who described the feedback or disturbance as rotated or claimed the application of a rotational strategy. Nine out of 16 subjects in the implicit group were considered not to have awareness, and, interestingly, two out of eight explicitly instructed subjects were also classified as unaware. For the statistical analyses we used the same data as for the correlations, i.e. the first bin of the mean adaptation and clamp trail indices of both blocks and entered them into two ANOVAS with the factors Group (aware, unaware) and Rotation Size (20, 40, 60). Neither the analysis of adaptation index (effect of Group: p = 0.32) nor that of clamp trial index (effect of Group: p = 0.59) yielded any significant effects, showing that awareness measured by means of a questionnaire, in contrast to that measured by PDP, was not related to adaptation.

## Discussion

The aim of the present study was to find out whether the degree of awareness of the nature of the perturbation depends on its magnitude. An implicit group and a group of subjects using explicit strategies adapted to 20°, 40° and 60° cursor rotations, and we measured awareness and unawareness indices after each adaptation block with a process dissociation procedure. The analyses revealed a larger awareness index in the explicit than in the implicit group and a larger awareness index after adaptation to 60° than to 20° rotation for both groups. This did not depend on the order in which rotation sizes were presented to the subjects. Adaptation and clamp trial indices were also larger in the explicit than in the implicit group. Furthermore, initial adaptation measures—adaptation and clamp trial indices of the first bin—correlated to the size of awareness indices. Savings did not differ between groups and only showed a trend of being larger for larger rotation sizes. The explicit but not the implicit participants transferred savings from the first to the following rotation sizes. Finally, the analyses revealed that awareness measured by means of a questionnaire, in contrast to that measured by PDP, was not related to adaptation.

### Awareness

The degree of awareness of the nature of the perturbation did clearly depend on the perturbations´ magnitude in both groups. Naïve participants developed a negligible awareness index of 0.02 after adaptation to 20° and 40° and an awareness index of 0.13 after 60° rotation of visual feedback. This result is consistent with previous work showing awareness only in three out of 27 participants when a 30° rotation was introduced [1]. We can further conclude that awareness does not arise when visual rotations are introduced gradually in steps of 0.25° to 10° [17, 16, 20, 18]. Our results more generally suggest that the development of awareness depends on the size of target error, i.e. the perceived error between cursor and target. Since in gradual adaptation paradigms the size of perturbation steps are deliberately chosen to induce only small target errors, there should also be no awareness after gradual adaptation to optical shifts using prisms [58, 59, 13], to visuomotor gains [15], viscous force-fields [60, 17, 61, 19] or after gradual saccade adaptation [14]. The present findings thus confirm the notion that participants adapting to gradually introduced perturbations with very small increases of perturbation size are usually not aware [19].

Moreover, the outcome of the present study can help explaining the actual disagreement on whether the gradual adaptation paradigm can improve the amount of recalibration [14] as shown in several studies [14, 62, 13, 16, 15] but not in others [17, 63, 18]. Also, intermanual transfer was revealed after sudden but not after gradual adaptation by Malfait and Ostry (2004), whereas this difference was not reported in other studies [2, 32]. This could be due to the chosen perturbation size that left participants adapting to the sudden introduced distortion equally unaware as the ones in the gradual group. For example, rotation sizes of 22.5° to 32° were used in those studies that did not find a difference in retention or intermanual transfer [17, 32, 2]. To finally solve this disagreement, further studies should be performed that identify the actual size of perturbation leading to awareness on the one hand and test awareness and retention or intermanual transfer at the same time on the other hand. Of course, gradual and sudden adaptation differ not only with respect to awareness and have consequently been shown to be based on distinct neural correlates [64, 60, 18].

It can be argued, that the group difference in awareness is due to the group difference of the inclusion index, that is the lack of the implicit group of reproducing what was learned compared to the explicit group. However, underestimation of awareness in the implicit group is unlikely, because those factors that might have contributed to the increased loss between the end of adaptation and inclusion are rather implicit or unaware: the passage of time, reaching without visual feedback or the observed decrease of errors during ongoing reaching during no feedback trials [65–68].

We found no effect of the order of rotation sizes on the amount of awareness. However, we cannot rule out that a possible beneficial effect of previous adaptation to different rotation sizes was cancelled out by a detrimental effect due to fatigue or to forgetting the instructions in the explicit group. Future studies should be conducted with a between-subject design in which each group gets exposed to a single rotation size only.

### Unawareness

As expected the unawareness index was smaller in the explicit compared to the implicit group but with mean indices of approx. 0.55, 0.4 and 0.3 after adaptation to 20°, 40° and 60°, respectively, unawareness was still surprisingly large in explicitly instructed participants. On the one hand, this large proportion of unawareness might be consistent with the results of a study by Mazzoni and Krakauer (2006) in which participants used an explicit strategy to quickly reduce errors during adaptation to a visual rotation. Later on during learning, reaching errors increased again, representing a simultaneous implicit adaptation process driven by sensory prediction errors. Our unawareness index could reflect this implicit process. On the other hand, we could also be seeing a modulation of strategies over the time course of adaptation with different strategies being differently accessible to consciousness as has been suggested earlier [69].

### Explicit and implicit group

Comparison of adaptation of both groups revealed a larger initial adaptation index in the explicitly instructed group. The magnitude of early adaptation clearly correlated to awareness across groups and for all rotation sizes, whereas we found no correlation between late adaptation and awareness for 20° and 60° rotations. This pattern of findings is in line with the results of a previous study showing explicit instructions leading to increased early but not late adaptation [1]. Since the explicit group in the present study was a priori instructed and, thus, aware of the nature of the perturbation it can be assumed that awareness leads to increased initial adaptation and not vice versa increased initial adaptation leads to awareness. It should be noted that the increased AI of the first bin could be due to a greater reduction of errors during those first nine trials or to a larger initial AI as a result of instructions or savings. We do not have sufficient data to distinguish these possibilities. Contrary to our findings of increased clamp trial indices in explicitly instructed participants, reduced catch trial performance in an explicit compared to an implicit group was reported earlier [1]. While the explicit subjects were instructed to turn off their strategy during catch trials in that study, our clamp trials came without notice or instructions and, therefore, we probably measured the sum of multiple learning mechanisms. Instead of isolating recalibration, the same processes as during perturbed trials might have been present in the explicit group. Hence, it is not surprising that clamp trial indices, equivalent to adaptation indices, correlated to awareness for all rotation sizes.

Even though we found no significant effects of savings, there was a marked increase from almost no savings in the 20° adaptation to savings in 40° and 60° adaptation. This is in line with previous work showing savings after 45° but not after 30° rotation of visual feedback [57]. In the current study, explicitly instructed participants further transferred savings from the first to the following rotation sizes in contrast to the implicit group. To our knowledge, no study has yet tested savings after participants had used an explicit strategy. But the result of the implicit group is in agreement with uninformed subjects showing faster relearning of an unexperienced perturbation only when rotation sizes differed by 75° and not 45° [57] or when they differed in essence like the difference from left-right reversal to a 180° rotation [70]. Interestingly, awareness did not correlate with any of our savings measures.

Multiple learning processes have been suggested to facilitate motor behaviour during adaptation. A distinction has been drawn between slow and fast adaptation [71], error-based and reinforcement learning [72] or implicit and explicit processes [30, 22, 21, 69]. The present data does not allow any judgement on the overlap of different theoretical frameworks. But it can help disentangling awareness and explicit strategies, which have both been attributed to explicit processes [30]. Awareness or explicit knowledge of the nature of the perturbation is thought to be a prerequisite for explicit strategies [11, 30, 73]. The latter can either be evoked by instructions [1, 23, 22, 21, 25] or by color cueing [57]. Explicitly instructed participants showed increased initial adaptation and increased savings compared to the implicit group in the present study. But awareness only correlated to adaptation and not to measures of savings. This dissociation of results is supported by the notion that savings are reflected by model-free reinforcement of previously successful behavior [62] and are thus linked to action selection or re-aiming strategies [69, 57].

### Measuring awareness

The possibility to measure awareness as well as unawareness as an index is an evident advantage of using PDP over the use of a questionnaire that only allows the classification of aware or unaware. Moreover, questionnaires may underestimate awareness due to the difference of retrieval contexts or because knowledge is held with low confidence [41, 40, 38, 37, 39]. Unlike the awareness index measured by PDP, the results of the questionnaire in the present study were neither related to adaptation nor to clamp trial behaviour. These results suggest that awareness of the nature of the perturbation can be more closely captured using PDP.

Heuer and Hegele measured explicit knowledge by providing the participants with a line which they moved through verbally instructing the experimenter until they found it to match the direction of a successful hand movement [12, 11, 45]. Yet another task was used by Taylor et al. (2014) to measure explicit learning. Here, participants reported their aiming direction with the help of a circular array of landmarks encircling the target. Both methods use predictions during the same sort of task and might therefore, as outlined in the introduction, be based on feelings of familiarity [47] and lead to an overestimation of awareness [74]. Further research is required to compare the results of those prediction task methods to those of the PDP.

## Conclusions

In conclusion, the results of this study suggest that the development of awareness of the nature of the perturbation depends on its size and confirm the idea that participants adapting to gradually introduced perturbations are usually not aware. Moreover, our findings can help explain the disagreement regarding the effects of the gradual adaptation paradigm by proposing that some studies chose perturbation sizes which left participants adapting to the suddenly introduced distortion just as unaware as those in the gradual group. Furthermore, the present results emphasize the importance of controlling or monitoring awareness in future studies comparing gradual and sudden adaptation. The awareness index of the current study measured by PDP correlated to the size of early adaptation, whereas the results of a questionnaire were not related to adaptation. To sum up, our results can thus explain the contradiction of previous studies analysing the effect of cognitive components such as explicit strategies and explicit knowledge, i.e. awareness, on sensorimotor adaptation.
